# French women’s knowledge of and attitudes towards cervical cancer prevention and the acceptability of HPV vaccination among those with 14 – 18 year old daughters: a quantitative-qualitative study

**DOI:** 10.1186/1471-2458-12-1034

**Published:** 2012-11-27

**Authors:** Julie Haesebaert, Delphine Lutringer-Magnin, Julie Kalecinski, Giovanna Barone, Anne-Carole Jacquard, Véronique Régnier, Yann Leocmach, Philippe Vanhems, Franck Chauvin, Christine Lasset

**Affiliations:** 1 Université Lyon 1, CNRS UMR 5558 Centre Léon Bérard, 28, rue Laënnec, 69373 cedex 08, Lyon, France; 2 Institut de Cancérologie Lucien Neuwirth, CIC-EC 3 Inserm, IFR 143, Saint-Etienne, France; 3 Sanofi-Pasteur MSD, Lyon, France; 4 Université Lyon 1, CNRS UMR 5558 and Hospices Civils de Lyon, Lyon, France

**Keywords:** Papillomavirus, HPV vaccine, Cervical cancer prevention, Acceptability, Women, Mothers

## Abstract

**Background:**

In France, it is recommended that girls and women aged 14–23 are vaccinated against the human papillomavirus (HPV). However, French women’s knowledge of and attitude towards the vaccine has been little studied.

**Methods:**

Thirty-nine general practitioners, representative of those working in the large Rhône-Alpes region, offered a self-administered questionnaire on cervical cancer (CC) prevention to all 18–65 year-old women who came for consultation during June and July 2008. In addition, semi-structured interviews were undertaken with a sample of those who had daughters aged 14–18.

**Results:**

Of the 1,478 women who completed the questionnaire, only 16.9% mentioned HPV as the cause of CC, even though 76.2% knew of the vaccine. 210 women had daughters aged 14–18, and 32 were interviewed. Compared with the wider group, more of these women were aware of the HPV vaccine (91.4%). 44.8% knew the target population and 17.1% the recommended ages for vaccination. 54.3% favoured HPV vaccination; 37.2% were undecided and only 0.9% were opposed. The main barrier to acceptance was the recency of the vaccine’s introduction and concern about possible side effects (54.9%); 14.1% preferred to rely on their GP’s decision. Factors associated with acceptance of the HPV vaccine were having previously vaccinated a child against *pneumococcus* (OR=3.28 [1.32-8.11]) and knowing the target population for HPV vaccination (OR=2.12 [1.15-3.90]). Knowing the recommended frequency of Papanicolaou smear testing (Pap test) screening was associated with lower acceptance (OR=0.32 [0.13-0.82]).

**Conclusions:**

Few mothers are opposed to HPV vaccination. Factors associated with acceptability were knowledge about the vaccine, acceptance of other vaccines and, unexpectedly, lack of knowledge about the recommended frequency of Pap testing. On multivariate analysis, compliance with recommendations for Pap test screening and socioeconomic factors had no effect on views about HPV vaccination. Given that concern about possible side effects is the major barrier to wider acceptance of the HPV vaccine in France, GPs have a key role in providing information.

## Background

Cervical cancer (CC) is the tenth most common cancer among French women [1]. In 2011, an estimated 2,800 new cases were diagnosed, leading to 1,000 deaths [1]. The fight against CC involves two strategies: Papanicolaou smear testing (Pap test), and vaccination against the sexually transmitted Human Papillomavirus (HPV) responsible for nearly all cases of CC [2]. In France, Pap test screening is recommended every 3 years for women aged 25–65 [3] and its use since the 1970s has markedly decreased CC incidence (2.9% decrease per year between 1980 and 2005) and mortality (4.0% decrease per year). Nevertheless, adherence with Pap test screening recommendations is still insufficient: in the period 2006–2008, it was estimated that only 56.6% of 25–65 year old women had had the smear within the previous three years [3]. There are also major geographical and socio-economic disparities in take-up rate [4].

More recently, a new primary prevention tool has become available with the development of two vaccines [5;6] targeting the high-risk HPV 16 and 18 genotypes responsible for 70% of CC [2]. These vaccines were licensed in France in 2007 and their use is funded. French health authorities recommended HPV vaccination for girls reaching 14 years with catch-up vaccination for girls aged 15–23 within their first year of sexual activity [5]. Since parental consent is required for vaccination of adolescent girls, parents, and especially mothers, are key decision-makers and potentially a major source of information for their daughters [6].

Attitudes towards this new vaccine seem to be positive, with most studies on parental attitudes reporting acceptance rates above 60% [7-11]. Nevertheless, awareness about CC and HPV among women is limited [12]. Factors associated with the decision to vaccinate are complex and varied. Previous studies highlighted concerns about safety, but evidence on the influence of parental knowledge [10,13-15], the preventive health practices of the mother [8,13,16] and socio-cultural context [8,10,11,17,18] is conflicting. In a recent meta-analysis of studies dealing with parental attitudes towards HPV vaccination, Trim et al. showed that being concerned about the potential risk of cancer and believing their daughters might contract HPV and related diseases were drivers for HPV vaccination [19]. However, parents would prefer to vaccinate older children and those who were sexually active. Previous attitudes towards other vaccines predicted acceptance of HPV vaccination, and a physician’s recommendation was also a major factor. In addition, because HPV is sexually transmitted, parental acceptance of the vaccine raises issues, including the perceived risk of promoting risky sexual behaviour [19,20], which are broader and quite distinct from those raised by other vaccines [21].

Most previously published studies on CC and HPV awareness have been from North America: with the exception of the United Kingdom, little such research has been carried out in Europe [19]. Several factors specific to France make it a particularly interesting case study of attitudes towards and uptake of HPV vaccination. First, the target age for immunization (14 years and above) is relatively old compared with that in other countries. Secondly, the controversy in the 1990s over the supposed link between the vaccination of adolescents against hepatitis B and the development of multiple sclerosis was sufficient to bring a halt to the immunization campaign. This particular scare about vaccination did not occur in other countries but may have had long-term effects on the perceived safety of mass vaccination in France. Such a finding would have wide implications. Finally, the fact that national regulators recommend HPV vaccination and the 65% reimbursement of costs could provide a favourable setting.

The first objective of this study, which combined quantitative and qualitative techniques, was to assess knowledge about CC, the Pap test and HPV vaccination in 18–65 year-old French women one year after the introduction of the vaccine. The second objective was to assess mothers’ acceptance of HPV vaccination for their 14–18 year old daughters and determinants of that acceptability. The age range 14–18 was chosen to encompass girls above the age at which vaccination is recommended in France and below the age at which they can themselves legally assent to the procedure. This study was part of the REMPAR (Recherche et Evaluation des Moyens de Prévention Anti-HPV en Rhône-Alpes) programme aimed at evaluating means of preventing HPV-mediated disease.

## Methods

This cross-sectional study, conducted in the Rhône-Alpes region of France in June and July 2008, used both quantitative (self-administered questionnaire) and qualitative methods (semi-structured interviews).

### Population

The study population was women aged 18–65 living in the Rhône-Alpes region who visited a participating general practitioner (GP) within the two months specified above, without exclusion criteria. Participants were consecutively recruited by GPs during a consultation (which can have been for any reason). The 39 physicians who agreed to participate were volunteers from a sample of 279 GPs who took part in an earlier study [22]. They were representative in gender, location and the nature of their practice of GPs in the Rhône-Alpes. This region has 6 million inhabitants and comprises 10% of the French population.

### Questionnaire data

GPs offered the self-administered questionnaire to all women who met the inclusion criteria (There were no exclusion criteria.). The anonymous questionnaire had five parts: 1) socio-demographic data (age, place of residence, occupation, educational level, marital status, number, age and gender of children), 2) practices related to disease prevention (immunization for themselves and their children, tobacco consumption), 3) gynaecological history (surgery, sexually transmitted diseases, history of Pap test screening and any abnormal findings), 4) knowledge about CC including its cause, the role of HPV, CC prevention (the role of the Pap test and HPV immunization) and 5) the acceptability of HPV vaccination.

Questions on socio-demographic variables, preventive health practices and gynaecological history were multiple-choice. Questions on knowledge were multiple-choice in relation to the Pap test and open-ended with regard to the cause of CC and understanding of HPV vaccination. Answers to open-ended questions were recoded according to predefined categories. The acceptability of HPV vaccination was assessed by asking respondents to choose a single response from six options (Table 1). Based on their answer, respondents were classified as favourable, undecided or opposed to HPV vaccination. The comprehensibility of the questionnaire was validated before its use in the survey through a pilot study involving three focus groups of 12 women each from low, medium and high socio-economic groups).

**Table 1:** Acceptability of HPV vaccination: options presented in the self-administered questionnaire, and the coding of responses

**About this vaccination against cervical cancer, if you have a daughter**	
1. I will get some information and consider it	Undecided
2. I prefer to wait	Undecided
3. She(they) is(are) already vaccinated	Favourable
4. I intend to vaccinate my daughter(s) in the future	Favourable
5. I will vaccinate my daughter(s) if she(they) asks me	Undecided
6. I think that this vaccination is useless	Opposed

### Qualitative data

Women completing the questionnaire who had daughters aged 14–18 were also asked if they would volunteer to take part in a semi-structured, face to face interview conducted in their own home by a sociologist. It was thought particularly important to understand the opinions, and the reasons underlying them, of mothers from an underprivileged, lower socioeconomic background, and of women whose questionnaire responses showed them to be opposed to HPV vaccination. Among the volunteers, selection for interview was designed to include a high proportion of mothers from both groups. Interviews explored in greater depth topics covered in the questionnaire, notably gynaecological, history, practices related to disease prevention, women’s understanding of HPV vaccination, and factors related to its acceptability. There was particular emphasis on the latter, given our concern to better understand the drivers of and barriers to acceptance. Interviews lasted 30 to 60 min, were audio-taped and were transcribed verbatim. A content analysis was carried out using an analysis grid designed to build on the topics addressed in the quantitative part of the study and explore them in greater depth. The results were then compared with analysis using specific software, NVivo (QSR International) according to the methodology proposed by Miles & Huberman [23].

### Statistical analysis

Descriptive data were calculated for the survey population as a whole and for the subgroup composed of mothers of one or more 14–18 year old girls. The relationship between mothers’ views (favourable *versus* undecided or opposed) and potential predictive factors was studied using Chi-square or Fisher's exact test for qualitative variables and using Student’s t or Mann–Whitney *U* test for quantitative variables. A stepwise backward logistic regression was used to determine the most suitable model for multivariate analysis. Variables with a p value ≤ 0.20 in univariate analysis were entered in the model and a p value ≤ 0.05 was considered statistically significant. Data analysis was performed using the SAS 9.1 software (SAS Institute, Cary, NC).

### Ethics

This study was approved by the French National Committees for personal data protection in medical research and conformed to the Declaration of Helsinki.

## Results

### The population studied

A total of 1,478 women completed the questionnaire: 702 (47.5%) were 18–39 years old and 776 (52.5%) aged 40–65 years. This age distribution is similar to that for the Rhône-Alpes region as a whole, for which the corresponding figures are 47.2% and 52.8% (p=0.81). Among these women, 210 (14.2%) had one or more daughters aged between 14 and 18 at the time of the study. Of these mothers, 32 were interviewed.

The socio-demographic characteristics, immunization status and gynaecological history of the study population as a whole and of the subgroup with daughters of vaccination age are given in Table 2. The mean (+/− SD) age for respondents overall was 40.5 +/− 12 years and 43.5 +/− 4.4 for the subgroup of mothers of teenage girls (in which, understandably, 40–49 year olds were over-represented). Except for a lower hepatitis B vaccination rate (35% for mothers compared with 51% for the sample as a whole), which was attributable to their age distribution, there were few differences in vaccination status, demographics or gynaecological history between the subgroup with daughters of vaccination age and respondents as a whole.

**Table 2:** Sociodemographic characteristics, vaccination status and gynaecological history of women as a whole and of mothers of 14–18 year old daughters

**Characteristic**	**Whole population**	**Mothers of a 14–18 yr old daughter**	**Interviewed mothers**
**N (%)**	**(N=1,478)**	**(N=210)**	**(N=32)**
18-29	309 (20.9)	-	-
30-39	393 (26.6)	40 (19.1)	9 (28.1)
40-49	414 (28.0)	150 (71.4)	21 (65.6)
50-65	362 (24.5)	20 (9.5)	2 (6.3)
Employment			
In employment	942 (69.6)	159 (79.5)	17 (53.2)
Unemployed/Housewife/Retired	411 (30.7)	41 (20.5)	15 (46.9)
Educational level			
Studies ongoing	100 (7.5)	1 (0.5)	-
Lower secondary	540 (40.6)	99 (52.1)	24 (75.0)
Upper secondary, non tertiary	606 (45.6)	81 (42.6)	8 (25.0)
Tertiary	83 (6.2)	8 (4.2)	-
Social/financial assistance^1^	67 (4.9)	6 (3.1)	5 (15.6)
Marital status			
Married/Living with a partner	1034 (70.5)	178 (86.0)	22 (68.8)
Single/Divorced/Widowed	431 (29.5)	29 (14.0)	10 (31.3)
Vaccination status themselves			
Diphtheria-Tetanus-Poliomyelitis and BCG	1376 (93.1)	200 (95.2)	32 (100.0)
Measles, Mumps and Rubella	714 (48.3)	86 (40.9)	9 (28.1)
Hepatitis B	754 (51.0)	74 (35.2)	11 (34.4)
Vaccination status of their children^2^			
Diphtheria-tetanus-poliomyelitis and BCG vaccine	1 096 (96.1)	205 (97.6)	32 (100.0)
Measles, Mumps and Rubella	837 (73.4)	172 (81.9)	21 (65.6)
Chickenpox	108 (9.5)	20 (9.5)	2 (6.3)
Rotavirus	10 (0.9)	0 (0.0)	-
Pneumococcus	257 (22.5)	38 (18.1)	5 (15.6)
Hepatitis B	568 (49.8)	103 (51.8)	16 (50.0)
Current cigarette smoker	248 (16.8)	33 (16.6)	6 (18.8)
Usual frequency of gynaecologic follow-up			
Each year	907 (63.6)	144 (70.6)	18 (56.3)
Every 2–3 years	305 (21.4)	38 (18.6)	7 (21.9)
Less than every 2–3 years/Never	213 (14.9)	22 (10.8)	7 (21.9)
Pap test within the last 3 years	1186 (82.9)	180 (87.0)	26 (81.3)
History of abnormal Pap test	147 (9.9)	27 (13.1)	4 (12.5)
Gynaecologic surgery	161 (10.9)	21 (10.7)	3 (9.4)
Sexually transmitted diseases	86 (5.8)	17 (8.1)	2 (6.3)

^1^In receipt of free health insurance or financial assistance.

^2^N= 1140 women who had at least one child.

### Knowledge about CC and its prevention

Among the 1,478 respondents as a whole, knowledge about the Pap test was quite good (61% knew its role), and better than that about the causes of CC: only 16.9% of women mentioned HPV in this context. However, the question on the causes of CC was open-ended while that on the Pap test was multiple-choice. Awareness of the HPV vaccine was high (76.2%) but the recommended target population and age for vaccination were not precisely known (Table 3). The media were the respondents’ major source of information on HPV vaccination: 54.7% of women had heard of the vaccine through television while only 16.0% said they had been informed about the vaccine by their physician.

**Table 3:** Knowledge about cervical cancer and its prevention among women and mothers of 14–18 year-old daughters

**Question**	**Whole population**	**Mothers of a 14–18 yr old daughter**
	**(N=1,478)**	**(N=210)**
What is the role of the Pap test? ^*1*^		
Answer correct : to prevent CC	901 (61.0)	137 (65.2)
Incorrect: to treat CC, to prevent all gynaecologic cancers, to check the ovaries	338 (22.8)	50 (23.9)
No information/No response	239 (16.2)	23 (10.9)
When should a woman have a Pap test? ^*1*^		
During her whole adult life	1 211 (81.9)	178 (84.8)
Incorrect : before or after the menopause	87 (5.9)	10 (4.8)
No information/No response	180 (12.2)	22 (10.4)
How often should she have a Pap test? ^*1*^		
Every 2–3 years (French national recommendation)	590 (39.9)	112 (53.3)
Yearly	795 (53.8)	91 (43.3)
Incorrect : once or from time to time	18 (1.2)	0 (0.0)
No information/No response	75 (5.1)	7 (3.3)
What is the cause of CC?^2^		
HPV	251 (16.9)	44 (20.9)
Related response (STD, viral infection)	120 (8.1)	22 (10.5)
Incorrect cause mentioned	23 (1.5)	11 (5.2)
No information/No response	1084 (73.3)	133 (63.3)
Have you ever heard of HPV vaccination? ^*1*^		
Yes	1127 (76.2)	192 (91.4)
Who should be vaccinated?^2^		
Young girls before or within a year of first intercourse	327 (22.1)	64 (44.8)
Answer nearly correct^3^	220 (14.9)	30 (14.2)
Incorrect	447 (30.2)	82 (39.0)
No information/No response	484 (32.8)	34 (16.2)
At which age is vaccination recommended?^2^		
14 -23	180 (12.2)	36 (17.1)
Answer close to recommendation: (*14*–*23 +/− 4 years*)	612 (41.4)	91 (43.3)
Incorrect	135 (9.1)	40 (19.0)
No information/No response	551 (37.3)	43 (20.5)

*CC*, Cervical cancer, *Pap test*, Papanicolaou smear test, *HPV*, Human papillomavirus.

^1^Multiple Choice Question.

^2^recoded open-ended question.

^3^reply included only one of the two concepts: young girls or before/within the first year after the first intercourse.

Knowledge about the Pap test among mothers of 14–18 year old daughters was not different from that of the wider population of women surveyed (65.2% vs 61.0% knew its role). As with the wider sample, relatively few mothers of teenage girls mentioned HPV as a cause of CC (20.9% vs 16.9%). However, during the interview, without multiple-choice options, mothers had more difficulties than the wider group in explaining the role of Pap testing: many said “*It is for prevention*” without specifying the disease that was being prevented. The mothers who knew about HPV in the interview were mainly those who had already vaccinated their daughters. These women had also had the situation explained by their GPs before vaccination. Even so, many were not clear about the link between HPV infection and CC. All but one of the 32 mothers interviewed (91.4%) knew about the HPV vaccine. As in the wider population of women, mothers had heard of this vaccine mainly through television (56.2%), but a greater proportion (39.1% vs 16.0% for respondents as a whole) had had information from their physicians.

Despite this, more than a half of the mothers did not know the target population or the precise recommended ages for vaccination (although most responses such as “young women” and “adolescents” were broadly correct). In interviews, they explained “*It is for teenagers*”, without mentioning ages. They knew from their physicians who should be vaccinated “*My physician told me that this concerned my daughter*”, because of her age and sexual history. However, few mothers had a detailed recollection of the information provided.

### Acceptability of the HPV vaccine among mothers of 14–18 year old daughters

Among the 210 mothers who completed the questionnaire, 194 responded to the question regarding HPV vaccine acceptability: 2 were opposed (1.0%), 78 were undecided (40.2%) and 114 were favourable (58.8%). A total of 46.4% had already vaccinated their daughters (Table 4). Among the 32 mothers interviewed, 2 were opposed to the vaccine, 8 were undecided and 22 were favourable. The latter group included the 13 who had already vaccinated their daughters.

**Table 4:** HPV vaccine acceptability among mothers of 14–18 year old daughters (N=210)

**Position**	**N (%)**
Favourable	114 (54.3)
My daughter(s) is/are already vaccinated	53 (25.2)
I intend to vaccinate my daughter(s) in the future	61 (29.1)
Unfavourable (Undecided/Opposed)	80 (38.1)
I will get some information and consider it	41 (19.5)
I prefer to wait	22 (10.5)
I will vaccinate my daughter(s) if she(they) asks me	15 (7.1)
I think that this vaccination is useless	2 (0.95)
Missing data	16 (7.6)

The major reason given in interviews by favourably disposed mothers (and cited by 65.8%) was that vaccination offered the opportunity of preventing a severe and potentially fatal disease, namely CC, in their daughters. The perceived danger of CC was frequently mentioned as driving the decision to vaccinate. Mothers mentioned the “*fear of cancer*” and their wish to “*protect* (their) *child*”. One explained: “*I don’t want my daughter to tell me ‘I have a cervical cancer’ while a vaccine exists*”. The second most frequent reason for vaccination (reported by 10% in questionnaires and 40% in interviews) was the favourable opinion of the physician. Those interviewed explained that physicians reassured them if they had any questions: “*She recommends it. I think I can trust her. She told me if she had a daughter of this age, she would vaccinate her against HPV.*” Nevertheless, in the questionnaires, 14.4% of favourably disposed mothers mentioned incorrect expectations of the vaccine such that it would eradicate risk of all gynaecological cancers or prevent all sexually transmitted diseases (STDs).

Undecided and opposed mothers justified their position by the fact that we have little experience of the vaccine to look back on and by fear of side effects with what is a new vaccine (such reasons were cited by 54.9% in questionnaires). Some mothers interviewed had in mind the controversy over a possible link between hepatitis B vaccination and multiple sclerosis which affected France after a mass hepatitis B immunization campaign among adolescents in 1994. Another 14.1% preferred to rely on their physician’s decision and waited to know his opinion, and 7.0% preferred to let their daughters decide by themselves. The key role played by physicians is illustrated by a mother opposed to the vaccine who explained in an interview that she did not want to vaccinate her daughter because her GP was against it: “*He told me not to vaccinate my daughter*”. Mothers from low socio-economic background who were interviewed seemed to adhere totally to their physicians’ opinion while those in higher professional categories wanted to know his opinion but had a more critical point of view.

In questionnaires, other concerns mentioned related to sexual issues: 5.6% found it difficult and too early to have a discussion about sexuality with their adolescent daughters, fearing that this might encourage sexual activity. Others preferred good gynaecological follow-up with regular Pap tests (4.5%). The cost of the vaccine was not mentioned by mothers.

### Factors among mothers that appeared to influence their decision about HPV vaccination

Analysis of factors associated with a favourable *versus* undecided/opposed opinion are presented in Table 5. In the multivariate model, mothers favourable towards the HPV vaccine were more likely than those who were not to have already vaccinated their child against *pneumococcus*. They were more aware of the target population for HPV vaccination but less knowledgeable about how frequently women should have Pap test screening. Educational level, tobacco consumption and history of STDs were excluded from the final model.

**Table 5:** Factors associated with HPV vaccination acceptance among mothers of 14–18 year old daughters (N=194), univariate and multivariate analysis

**N (%)**	**Favourable (N=114)**	**Undecided opposed (N=80)**	**p value**	**Crude OR**	**95% CI**	**Adjusted OR**	**95% CI**	**p value**
Age^1^								
*≥* 40 years old	88 (77.2)	69 (86.3)	0.114	1				
< 40 years old	26 (22.8)	11 (13.8)		1.85	0.56-4.01			
Employment								
Unemployed/Housewife/Retired	24 (18.2)	21 (32.4)	0.640	1				
In employment	90 (81.8)	59 (77.6)		1.33	0.68-2.60			
Social/financial assistance	5 (4.59)	1 (1.4)	0.234	3.62	0.41-31.59			
Family situation								
Married/Living with a partner	95 (85.6)	68 (85.0)	0.982	1				
Single/Divorced/Widowed	19 (14.4)	12 (15.0)		1.13	0.51-2.48			
Vaccination status themselves								
Diphtheria-Tetanus-Poliomyelitis and BCG	111 (97.4)	75 (96.1)	0.630	2.47	0.57-10.65			
Measles, Mumps and Rubella	41 (37.3)	36 (48.0)	0.339	0.69	0.39-1.24			
Hepatitis B	41 (37.3)	31 (41.3)	0.386	0.89	0.49-1.61			
Vaccination status of their children								
Diphtheria-tetanus-poliomyelitis and BCG vaccine	113 (99.1)	78 (97.5)	0.367	2.90	0.26-32.54			
Measles, Mumps and Rubella	97 (85.1)	65 (81.3)	0.478	1.32	0.62-2.83			
Chickenpox	13 (11.4)	7 (8.8)	0.798	1.34	0.51-3.52			
Pneumococcus^1^	27 (23.7)	7 (8.85)	0.007	3.24	1.33-7.86	3.28	1.32-8.11	0.010
Hepatitis B	57 (52.3)	39 (51.4)	0.991	1.05	0.59-1.86			
Current smoker^1^	22 (20.8)	7 (9.09)	0.033	2.62	1.06-6.49			
Educational level^1^								
Tertiary	15 (14.3)	17 (24.6)	0.143	1				
Upper secondary/non tertiary	35 (33.3)	16 (23.2)	0.051	2.48	0.996-3.99			
Primary/lower secondary	55 (52.4)	36 (52.2)	0.185	1.73	0.77-3.90			
History of STD^1^	7 (6.1)	9 (11.2)	0.203	0.52	0.18-1.45			
Yearly gynaecological follow up	76 (68.5)	57 (73.1)	0.602	0.81	0.44-1.51			
Most recent Pap test within the past three years	101 (90.2)	67 (84.8)	0.262	1.51	0.66-3.46			
“Pap test aims to prevent CC”	76 (66.7)	54 (67.5)	0.903	0.96	0.52-1.76			
“Pap test should be performed during the whole of adult life” ^1^	91 (79.8)	73 (91.3)	0.030	0.38	0.15-0.93	0.32	0.12-0.82	0.018
Have heard of HPV vaccine	112 (98.2)	69 (87.3)	0.004	8.12	1.13-38.1			
“HPV vaccine is recommended for young adolescent girls before sexual debut” or answer close to this” ^1^	60 (52.6)	30 (37.5)	0.038	1.85	1.03-3.32	2.12	1.15-3.90	0c016

^1^included in multivariate model, *OR*, odds ratio, *CI*, confidence interval, *CC*, cervical cancer, *cpo*, sexually transmitted disease.

## Discussion

One year after the licensing of the HPV vaccine in France, knowledge about the role of the Pap test in CC prevention is quite good. However, when asked an open-ended question about the cause of CC, the link between HPV and CC was not widely made. Even so, among mothers of 14–18 year old girls, the majority accepted the value of HPV vaccination -- although 37% were still undecided.

Responding to multiple choice questions, 93.7% of women knew that the Pap test should be conducted yearly or every 2–3 years and 82.0% answered correctly that screening should continue throughout adult life. However, only 61% answered that the purpose of Pap testing is to prevent CC. It seems that women passively accept the Pap test without really knowing why: 80% of French women are reported to have Pap tests on the advice of their physician and not on their own initiative (cervical screening in France is opportunistic.) [24]. Our data suggest that the cause of CC is not widely known. Despite information campaigns on CC and its prevention related to the marketing of HPV vaccines, few respondents (17% of the wider population of women studied and 21% of mothers) spontaneously mentioned HPV. Nevertheless, this level of awareness is much higher than before introduction of the vaccine: in surveys reported in 2001 and 2004, fewer than 2% of women mentioned HPV as the cause of CC [25,26].

In contrast, the HPV vaccine itself is now well known: 76% of our overall sample and 91% of mothers had heard of it. However, they did not know precisely who should be vaccinated or at which age. This may be explained by the fact that the predominant source of information on the vaccine is the media (mentioned by 54.7%). Few women had been individually informed by their physicians.

The majority of mothers with a 14–18 year old daughter were supportive of HPV vaccination. A substantial proportion (38% in our sample) is still undecided, but very few (< 1%) are clearly opposed. Even if French physicians have also been shown to be supportive of HPV vaccination [22], the reality is that in France in 2010, only 38.7% of 14 year old girls had received at least one dose of the vaccine. The corresponding uptake among 16 year olds was 50.0%, and among 17 year olds 52.6%. HPV vaccine acceptance in our sample was lower than was suggested by data from 2007 when the proportion of parents intending to vaccinate their child was 67% [19]. The main issue remains the large population of undecided mothers, and we need a better understanding of factors influencing parental decisions. Protecting one’s daughter against a potentially lethal cancer is the main reason mothers give for vaccinating their daughters. Efficacy is not questioned, and the main concern with HPV immunization is the newness of the vaccine. A year after the vaccine’s introduction, mothers still feared the emergence of unexpected side effects. Vaccine safety is frequently the main concern about HPV immunization reported in the literature [10,17,26,27]. In our interviews, mothers who were still to make up their minds linked their reluctance to accept the new vaccine to the controversy in France about the suggested connection between Hepatitis B vaccination and multiple sclerosis [28].

Physicians represent a key influence [29], and their advice is crucial in avoiding misconceptions among undecided mothers [7,17,27]. Freed et al. showed that the principal information source that mothers trusted was their physician (76%) [30] and Little et al. found that mothers who received information from their health care provider were significantly more likely to intend to vaccinate their daughters than those who did not receive such information (OR=3.56 [1.52-8.45]) [31]. Along with others, our data suggest that provision by physicians of more complete information on vaccine safety could encourage acceptance [10,13,32]. As a specific example of this, we found that mothers who knew the target population for the HPV vaccine were more favourable towards it.

We also identified other determinants. Having already vaccinated their children against *pneumococcus* was associated with a positive attitude towards HPV vaccination. This may reflect a wider attitude towards vaccination. Mothers who believe vaccines in general are safe are more willing to vaccinate their daughters against HPV [10,33-35]. More surprisingly perhaps, our findings suggested that mothers who did not know how frequently Pap testing should take place were more favourable towards HPV vaccination than those with greater knowledge. A possible explanation is that women who are more aware of Pap testing regard it as an effective method to prevent CC and so see less need for vaccination. In justifying their position, some mothers unfavourable to the vaccine argued that an effective method to prevent CC already existed.

However, on multivariate analysis, Pap test adherence itself was not associated with less acceptance of the vaccine.

Socio-economic characteristics were not associated with HPV vaccine acceptance. Unfavourably disposed mothers did not mention cost as a barrier to vaccination in either the questionnaire or interviews. This may be due to the partial reimbursement (65%) by French public health insurance.

Our results do not support the concern raised previously that underprivileged populations or populations with low adherence to Pap test screening would be less accepting of HPV vaccination. With regard to educational level, the results tended to be the reverse, with a lower acceptance of HPV vaccination among better educated mothers, as has previously been found [10,11,17,27]. However, two factors found relevant in a recent review were little mentioned by mothers in our study [19]. Our respondents did not express the fear that vaccination would encourage more risky sexual activity, and they did not reflect a belief that the age for vaccination was too young [19]. With respect to lack of concern about potential sexual disinhibition, our findings are in line with those of the recent Canadian study by Ogilvie et al. [28]. And in relation to the age recommended in France for HPV vaccination, it is worth noting that this is in fact older than in many countries [36]. Since the median age at first intercourse in France is 17, many girls aged 14–18 will already have begun sexual activity, while others are close to doing so.

Possible limitations in our findings should be mentioned. Certain factors previously shown to be associated with HPV vaccine acceptance, such as the younger age of mothers, STD history, tobacco consumption and educational level, were significant in univariate analysis but not after multivariate adjustment. A lack of power due to the relatively small sample of mothers could explain these discrepancies. Another limitation is that since recruitment took place during a GP consultation our respondents may have been more attuned than the wider population to their medical needs. Women included had a history of good gynaecological follow-up and a high rate of Pap test screening: 82.9% of the women sampled (and 87.0% of mothers) had had their most recent Pap testing within the past three years. Data from the French public health insurance system estimate that Pap test coverage nationally is only 56.6% [3]. However in a more recent study, 79.7% of a sample of 1,688 20–65 year old women reported having had a Pap test in the past three years [37], and our sample’s characteristics seem to be representative of women in the Rhône-Alpes region with a similar age distribution.

Our choice of question to assess the acceptability of HPV vaccination, and the multiple choice responses offered, could also be debated. Our intention was to cover the range of mothers’ attitudes and behaviour. Focus groups prior to the study showed a good understanding of this question and of the options for response. Moreover, all respondents were able to justify their opinion and give further detail in an open ended question; and those interviewed had additional opportunities to discuss why they were favourable towards vaccination, or otherwise.

Finally, we used a cross-sectional design. This did not allow us to determine whether the associated factors identified are the cause or the consequence of HPV vaccine acceptance: greater knowledge about the vaccine could be the result of information given during its administration or, alternatively, might have driven the decision to seek vaccination.

One year after French national health authorities recommended HPV vaccination, knowledge and awareness about HPV as the cause of CC is still poor a mothers of girls in the targeted age range are globally favourable towards the HPV vaccine but are seeking additional information. The results of this study suggest the type of information that could be disseminated to improve vaccine uptake. This relates principally to the record of vaccine safety. However, women do not fully understand the place of HPV vaccination alongside Pap testing in CC prevention because they are not fully aware of HPV as the cause. The need to complete this link in the information chain could also be addressed. Appropriate information on these topics may improve mothers’ acceptance of vaccination for their daughters. Physicians seem best placed to answer their questions.

## Conclusions

Women know that the aim of Pap testing is to prevent CC; and they know that there is an HPV vaccine. But they do not know that HPV causes CC. The majority of mothers of 14–18 year old daughters were favourable towards HPV vaccination. Those who were unfavourable justified their opinion mainly by the fear of side effects. Factors associated with acceptability were knowledge about the HPV vaccine, acceptance of other vaccines and lack of knowledge about the recommended frequency of Pap testing. Compliance with recommendations for Pap test screening and socioeconomic factors did not significantly affect views on HPV vaccination. GPs seem to have a key role in providing further information about HPV vaccination (particularly in relation to the virus as the cause of CC) and in reassuring women of its safety.

## Abbreviations

CC: Cervical cancer; GP: General practitioner; HPV: Human Papillomavirus; OR: Odds ratio; STD: Sexually transmitted disease.

## Competing interests

Authors declared no competing interests expect Yann Leocmach and Anne-Carole Jacquard who are employees of Sanofi Pasteur MSD, Lyon, France.

## Authors’ contributions

JH: performed the statistical analysis and interpretation of data, wrote the manuscript. DL-M: participated in the design of the study, helped to draft the manuscript and revised it critically for important intellectual content. JK: conducted and interpreted the interviews, wrote the qualitative part of the manuscript. GB: participated in the design of the study and coordinated the data collection. A-CJ: has given final approval of the version to be published. VR: participated in the design of the study. YL: helped in the interpretation of data and has given final approval of the version to be published. PV: participated in the design of the study. FC: participated in the design of the study and helped in the interpretation of data. CL: participated in the design of the study and its coordination and helped in the interpretation of data; revised the manuscript critically for important intellectual content. All authors read and approved the final manuscript.

## Pre-publication history

The pre-publication history for this paper can be accessed here:

http://www.biomedcentral.com/1471-2458/12/1034/prepub
